# Unveiling the life of archaea in sediments: Diversity, metabolic potentials, and ecological roles

**DOI:** 10.1002/imo2.56

**Published:** 2025-01-28

**Authors:** Dayu Zou, Yanling Qi, Jinjie Zhou, Yang Liu, Meng Li

**Affiliations:** ^1^ Archaeal Biology Centre, Synthetic Biology Research Center, Shenzhen Key Laboratory of Marine Microbiome Engineering, Key Laboratory of Marine Microbiome Engineering of Guangdong Higher Education Institutes, Institute for Advanced Study Shenzhen University Shenzhen China; ^2^ College of Physics and Optoelectronic Engineering Shenzhen University Shenzhen China

**Keywords:** archaea, biogeochemical cycles, diversity, sediments

## Abstract

The domain Archaea was initially characterized as extremophiles upon its proposal. Recent significant discoveries have redrawn our views of archaeal biology, encompassing the identification of mesophilic archaeal groups, the expansion of archaeal diversity and metabolic capabilities, and the elucidation of evolutionary relationships among archaea, bacteria, and eukaryotes. Archaea are ubiquitous and constitute a substantial fraction of the microbial biomass within sediments. Therefore, comprehending their ecological roles is paramount for understanding their contributions to global geochemical cycles. In this review, we summarize the diversity of archaea across various sediment ecosystems, from terrestrial inland to deep‐sea environments, utilizing representative genomes supported by the Genome Taxonomy Database, which encompasses habitats such as hot springs, salt lakes, freshwater lakes, rivers, mangroves, estuaries, coastal regions, seafloor sediments, cold seeps, and hydrothermal vents. Furthermore, we integrate analyses of representative genomes with recent studies to highlight the metabolic potentials, novel enzymatic functions, and significant discoveries related to the carbon, nitrogen, and sulfur cycles across different archaeal lineages. Finally, we discuss recent research hotspots and achievements in archaeal studies, while projecting future exploration directions. The expanding diversity and metabolic capacities of archaea have broadened our perspective on the tree of life and underscored their critical impacts on ecosystems.

## Highlights

- Archaea are diverse and widespread across various sediment habitats.
- Archaea are vital contributors to element biogeochemical cycles in sediments.
- Some research hotspots and prospects for archaeal studies are proposed.

## Graphical abstract

Archaea exhibit extensive diversity and ubiquitous distribution in sediments across a range of habitats, from terrestrial inland to deep‐sea environments, including hot springs, salt lakes, freshwater lakes, rivers, mangroves, estuaries, coastal areas, seafloor sediments, cold seeps, and hydrothermal vents. The archaeal communities in sediments are predominantly composed of Thermoproteota, Halobacteriota, Asgardarchaeota, Thermoplasmatota, and Nanoarchaeota. Archaea play pivotal roles in the carbon, nitrogen, and sulfur cycles within sediments, with metabolic potentials varying across different archaeal lineages.

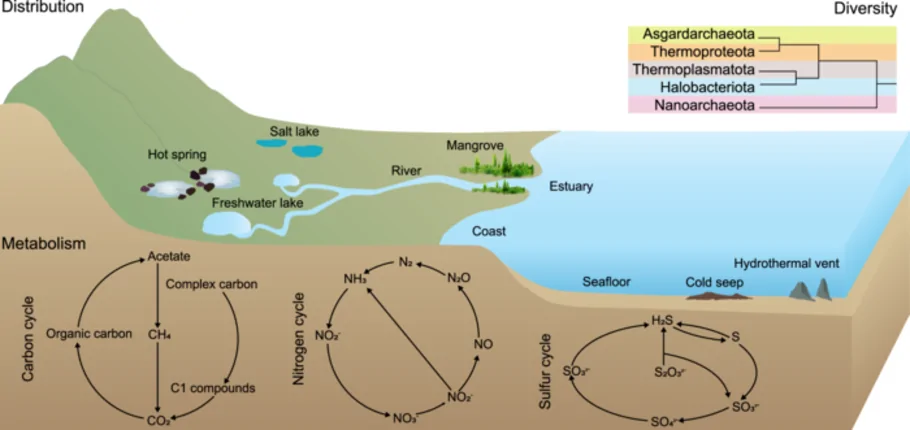

## 1 INTRODUCTION

Archaea, a diverse group of prokaryotes constituting a distinct domain of life, are differentiated from bacteria and eukaryotes in numerous aspects [1, 2, 3, 4]. In 1990, Woese et al. proposed the classification of archaea into a novel domain of life, the archaeal domain, alongside the bacterial and eukaryotic domains, based on nucleic acid‐based comparative and phylogenetic analyses [2]. Notably, archaea possess unique cell structures and genetic information processing systems that, while morphologically similar to bacteria, exhibit closer alignment with eukaryotes in terms of genome replication, transcription, translation, and other mechanisms of genetic information transfer [5, 6]. Initially, archaea were believed to predominantly inhabit extreme environments characterized by high temperatures and acidic‐alkaline conditions [7, 8]. However, the discovery of mesophilic archaeal groups has significantly broadened our understanding of archaeal distribution [9, 10, 11, 12], and their diverse metabolic potentials play a crucial role in driving various elemental cycles across different ecosystems [13, 14].

Sediments cover more than half of the Earth's surface, representing one of the largest microbial habitats on the planet, including significant populations of archaea. Recent estimates indicate that archaea comprise a substantial portion of the benthic microbial community, ranging from approximately 12.8% in pelagic sites to around 40.0% in marginal regions [15]. Archaeal inhabitants are found across successive sediment ecosystems, from inland areas and land‐ocean intersections to the open ocean. In this review, we have collected and reanalyzed 2063 representative archaeal genomes obtained from the GTDB. This data set encompasses 339 samples derived from hot springs, salt lakes, freshwater lakes, rivers, estuaries, coastal regions, mangroves, seafloor sediments, cold seeps, and hydrothermal vents. Among the 19 currently recognized archaeal phyla in the GTDB, representative genomes from 18 phyla have been reported in sediment environments, encompassing 48 archaeal classes. Based on the analysis of these 2063 archaeal representative genomes, we explore the diversity and ecological significance of sediment archaea, emphasizing their role in global geochemical cycles. We elucidate the widespread distribution of archaea and their presence in extreme environments, and also discuss their involvement in carbon, nitrogen, and sulfur cycles and their contributions to sediment ecosystems.

## 2 MISCELLANEOUS ARCHAEA LIVING IN SEDIMENTS

**FIGURE 1:**
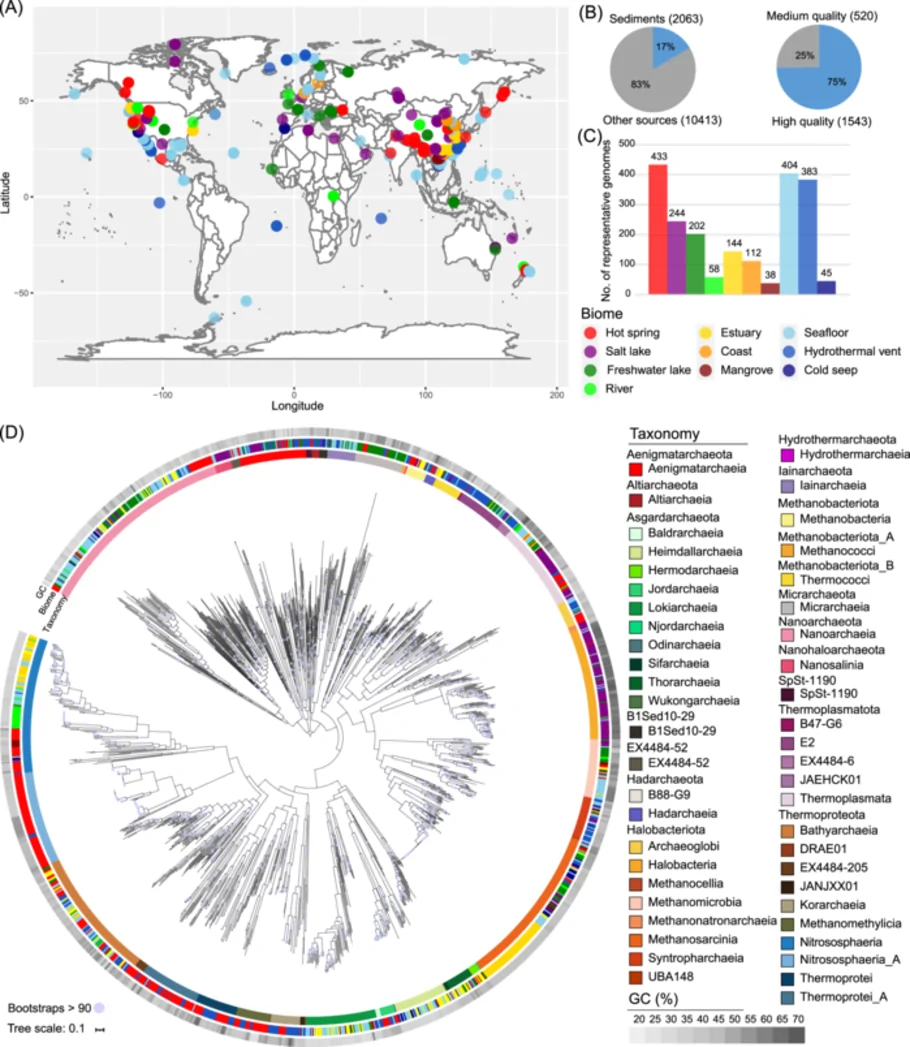
The overview of the geographical distribution of archaeal representative genomes. (A) Global location of sediment archaeal representative genomes. Colors represent different biomes. (B) The proportion of sediment archaeal representative genomes in the Genome Taxonomy Database (R220) and the percentage of high‐quality genomes used in this review. (C) The number of archaeal representative genomes from each biome. (D) The phylogenetic tree of all sediment archaeal representative genomes. Colors of the inner ring represent different archaeal clades at the class level. Colors of the middle ring represent the different biomes. The outer ring represents the GC content of each genome.

**FIGURE 2:**
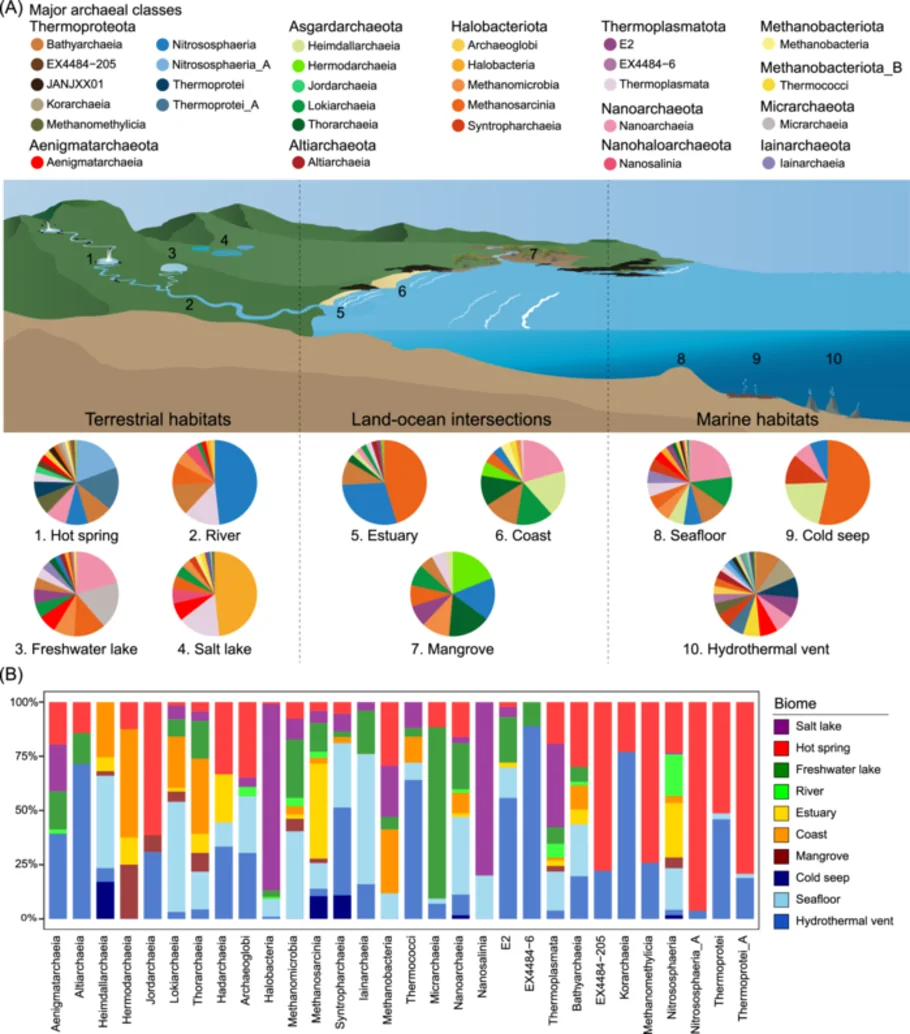
The community composition of archaea in different sediment habitats. (A) A schematic representation of the archaeal community composition at the class level in sediments from terrestrial habitats, land–ocean interfaces, and marine environments, based on the original sources of archaeal representative genomes. (B) The composition of representative genomes derived from various environments is displayed for each archaeal class.

In this review, we have compiled 2063 representative genomes across various sediment habitats (Figure 1A,B). The largest number of archaeal representative genomes has been identified in hot springs, seafloor sediments, and hydrothermal vent sediments, whereas the representative genomes in land–sea intersection zones are comparatively fewer (Figure 1C). Influenced by tidal movements and river discharges, a mix of terrigenous and oceanic archaea is found in these land–sea intersection areas, potentially leading to a reduced number of species‐level unique archaea in this region. The 2063 representative genomes utilized in this review span 18 phyla, predominantly affiliated with Thermoproteota, Halobacteriota, Asgardarchaeota, Nanoarchaeota, and Thermoplasmatota (Figure 1D). At the class level, taxonomic composition exhibits variability across different environments (Figure 2A). Certain lineages, including Methanosarcinia, Methanomicrobia, Nitrososphaeria, Bathyarchaeia, Thermoplasmata, Nanoarchaeia, Lokiarchaeia, and Thorarchaeia, are widely distributed from inland to marine sediments (Figure 2B). Conversely, other groups, such as thermophilic and halophilic extremophiles, are restricted to specific habitats. The following sections elaborate on the major archaeal groups found in different environments.

### Ubiquitous archaeal lineages on Earth

Ammonia‐oxidizing archaea (AOA), classified within the class Nitrososphaeria, are recognized as some of the most ubiquitous microorganisms found in terrestrial and marine aerobic sediments (Figure 2B). Their distribution and phylogeny have been extensively investigated through analyses of functional genes encoding ammonia monooxygenase subunit A (*amoA*) and 16S rRNA genes [16, 17]. The ecophysiology of these aerobic and autotrophic organisms has been comprehensively documented since the isolation of *Nitrosopumilus maritimus* SCM1, marking a significant advancement in understanding archaeal nitrification [18]. Studies focused on *amoA* genes have demonstrated that the abundance and distribution of AOA are closely related to environmental conditions, with lineages NS‐δ and NS‐γ (Nitrososphaeraceae) predominating in soil, NT‐α in freshwater sediments (*Ca*. Nitrosotaleales), NC‐α in hot springs (Nitrosocaldaceae), NP‐γ and NP‐α (Nitrosopumilaceae) in marine sediments [19]. This uneven detection frequency underscores the importance of adaptive strategies for their extensive phylogenetic diversity and cosmopolitan distribution.

Methanosarcinia, Methanomicrobia, and Methanobacteria are widely distributed methanogens frequently detected in anoxic sediments across terrestrial, land–sea intersectional, and marine ecosystems (Figure 2B), with the highest richness of these methanogenic lineages observed in estuaries [20]. The first archaeal isolate, a methanogen of order Methanobacteriales, was reported in 1958 [21]. Subsequently, diverse methanogen isolates contributed to the formation of class I (Methanococci, Methanopyri, and Methanobacteria) and class II (Methanosarcinia, Methanomicrobia, and Methanocellia) methanogenic lineages, which mainly utilize acetate, H_2_/CO_2_, and methyl compounds as substrates [22]. Recent studies have proposed three novel methanogenic groups: Methanomassiliicoccales (formerly RC‐III, now affiliated with the class Thermoplasmata) [23], Methanofastidiosa (formerly WSA2, now affiliated with the class Thermococci) [24], and Methanonatronarchaeia [25].

Bathyarchaeia, initially described from anoxic deep seafloor sediments, included several related groups that were later unified into a single lineage known as the Miscellaneous Crenarchaeotal Group (MCG) [26, 27]. Despite the lack of pure cultures or enrichments, investigations utilizing 16S rRNA genes and metagenomic analyses have unveiled the ubiquitous distribution and diverse metabolic potentials of these anaerobes [28, 29, 30, 31]. A recent study has classified 304 representative metagenomic assembled genomes (MAGs) of Bathyarchaeia into eight order‐level lineages with distinct environmental characteristics [32]. Evolutionarily, Bathyarchaeia are believed to have developed primarily towards freshwater environments from saline origins [33], indicating salinity as a key factor to the community composition and distribution. This habitat‐specific distribution underscores the diverse metabolic capabilities, ecological functions, and adaptation strategies of Bathyarchaeia in various sediment environments.

Members of the class Nanoarchaeia, including the Pacearchaeales and Woesearchaeales (formerly Deep‐sea Hydrothermal Vent Euryarchaeota Group 5 and 6, respectively), were initially found to be abundant in methane seep and hydrothermal vent sediments of the deep ocean [34, 35]. Subsequent studies have suggested that they rank among the most ubiquitous lineages across diverse terrestrial and marine sediment habitats [36, 37, 38]. A recent study highlighted the widespread occurrence of Woesearchaeales in freshwater sediments and coastal regions, categorizing them into ten subgroups primarily based on salinity [36]. Genomic insights reveal that most members of Nanoarchaeia exhibit characteristic features such as extremely small cell and genome sizes, alongside limited metabolic capacities.

The Asgardarchaeota, a taxonomically diverse and ubiquitous lineage, is distinguished by the presence of numerous eukaryotic signature proteins (ESPs) regulating processes such as cytoskeleton remodeling, signal transduction, nucleocytoplasmic transport and vesicular trafficking, and its close phylogenetic relationship with eukaryotes [39]. In the wake of the initial discovery of Lokiarchaeia (formerly Marine Benthic Group B, MBG‐B), a cadre of additional lineages has been delineated, including Thorarchaeia, Odinarchaeia, Heimdallarchaeia, Helarchaeales (which are classified within the Lokiarchaeia class), Gerdarchaeota (reclassified as the order JABLTI01 within the Heimdallarchaeia class), and Wukongarchaeia [39, 40, 41, 42]. As of 2023, the Asgard archaea have expanded to encompass 17 distinct lineages, spanning from the family to the class level [43, 44, 45, 46]. Within this diverse group, Lokiarchaeia and Heimdallarchaeia are notably prevalent in marine sedimentary environments, whereas Thorarchaeia demonstrates a more expansive ecological breadth, inhabiting lakes, estuaries, and seafloor sediments alike (Figure 2B) [47]. The recently discovered Helarchaeales and JABLTI01 are frequently detected in abyssal and coastal sediments, respectively [48]. The burgeoning diversity within the Asgard archaea lineage may not only illuminate the evolutionary trajectory of eukaryotes but also enhance our comprehension of their ecological roles.

### Archaeal extremophiles in extreme environments

Life on Earth persists across the full spectrum of salt concentrations encountered in both natural and anthropogenic habitats, from freshwater to saline and hypersaline conditions. Archaea play a significant role in the microbial communities of saline lakes, particularly in hypersaline lakes (salinity > 50.0 g/L) [49, 50]. For instance, in the Dong Taijinar salt lakes of the Qaidam Basin, with salinities surpassing 300 g/L, archaea account for over 95% of the metagenomic data, demonstrating their overwhelming dominance [51]. Archaea found in salt lakes are diverse (Figure 2A). The majority of known archaeal halophiles are phylogenetically affiliated with the class Halobacteria, which includes more than 357 species with validly published names as of 2023 [52]. Halophilic methanogens, primarily from the classes Methanosarcinia and Methanomicrobia, constitute a crucial component of the archaeal community in salt lakes, thriving at near‐saturation salt concentrations [53]. A recently discovered methyl‐reducing methanogen, *Methanonatronarchaeum thermophilum*, categorized from hypersaline lakes, is affiliated with the novel class Methanonatronarchaeia [54]. Additionally, the Nanosalinia group, formerly known as Nanohaloarchaeota, has been recognized in various salt lakes [55]. The continuous search for novel halophiles, such as the discovery of Halarchaeoplasmatales within the Thermoplasmata class [56], has expanded our understanding of the functioning and biogeochemical cycles of hypersaline ecosystems.

Terrestrial hot springs, globally distributed and well‐isolated habitats, support diverse microbial communities. The wide‐ranging physicochemical properties of hot springs, such as pH values ranging from −0.8 to 10.5 and temperature ranges from <10 to >100°C, significantly influence the composition of microbial communities [57, 58]. Archaea often dominate these communities, comprising extremophiles adapted to acidic, alkaline, and thermal conditions [59, 60]. Common archaeal lineages in terrestrial hot springs, as indicated by both 16S rRNA gene and metagenomic surveys, include Korarchaeia, Thermoplasmata, Thermoprotei, Archaeoglobi, and Thermococci [61, 62, 63, 64]. The diversity of archaea in hot springs has been further confirmed with the discovery of additional lineages, such as Aigarchaeota (currently classified as Caldarchaeales within the Nitrososphaeria_A class) [65], Geothermarchaeota (formerly known as Terrestrial Hot Spring Crenarchaeota Group, THSCG, now classified as Geothermarchaeales within the Nitrososphaeria class) [66], and Methanomethylicia (formerly named as Verstraetearchaeota) [67] (Figure 2A), enhancing our knowledge of thermophilic archaeal communities.

Marine sediments are repositories of a substantial microbial biomass. Archaea inhabit these sediments from the surface sulfate‐reducing layer to the deep methane‐producing zones, where they are estimated to constitute approximately 37.3% of all marine sedimentary microbes [15]. In contrast to the aerobic surface layer, which is dominated by the oxic lineage Nitrososphaeria, the anaerobic methane‐producing zone harbors a variety of anaerobic lineages adapted to deep environments [68]. While Bathyarchaeia and Asgardarchaeota are prevalent in anaerobic communities throughout the sediment depth, Hadarchaeia (formerly South‐African Gold Mine Miscellaneous Euryarchaeal Group, SAGMEG) and Theionarchaea (formerly Z7ME43, within the Thermococci class) are more abundant in deeper layers [68, 69]. Several recently reported lineages, such as Thermoprofundales (formerly Marine Benthic Group D and DHVEG‐1, within the E2 class) and Brockarchaeota (now classified as class EX4484‐205), are also type lineages widespread in subseafloor sediments [70, 71], indicating that marine benthic sediment serves as a reservoir for diverse archaea.

Hydrothermal vents are typically situated at global mid‐ocean ridges and seafloor‐spreading centers [72]. Hydrothermal sediments contain a variety of reduced sulfur compounds, organic compounds, and heavy metals [73, 74], representing a distinct ecological niche in the ocean. The microbial communities in these environments are predominantly composed of thermophiles and hyperthermophiles (Figure 2A), primarily from the classes Thermoprotei, Archaeoglobi, Thermococci, Methanosarcinia [72]. Another key lineage is Hydrothermarchaeia (formerly referred as marine benthic group E, MBG‐E), which was first reported as predominant in deep‐sea hydrothermal sediments [75] and is believed to survive in other non‐extreme environments [76]. Other lineages inhabiting hydrothermal vents include members of Bathyarchaeia, E2, and Nanoarchaeia (Figure 2A). Due to sampling limitations, the diversity of archaea in extreme and geographically isolated hydrothermal vents may exceed current understanding.

Marine cold seeps are typically located at the edges of continental shelves, characterized by sustained hydrocarbon fluid and methane gas seepage [77]. These chemosynthetic ecosystems harbor a rich diversity of archaea. Among the most prevalent lineages are the anaerobic methane‐oxidizing archaea (ANME), including ANME‐1 (class Syntropharchaeia) and ANME‐2 (class Methanosarcinia) (Figure 2A), which dominate the microbial community in cold seeps of the South China Sea and Atlantic margins [78, 79, 80]. ANMEs are also implicated in the syntrophic anaerobic methane oxidation process in anoxic freshwater and coastal sediments [81, 82, 83]. Moreover, genomes of Lokiarchaeia, Heimdallarchaeia, Bathyarchaeia, and Nanoarchaeia have been recovered from global cold seep sediment metagenomes [78, 84], suggesting a significant archaeal diversity in this unique ecosystem.

## 3 ARCHAEA ARE MULTI‐SKILLED IN SEDIMENTS

**FIGURE 3:**
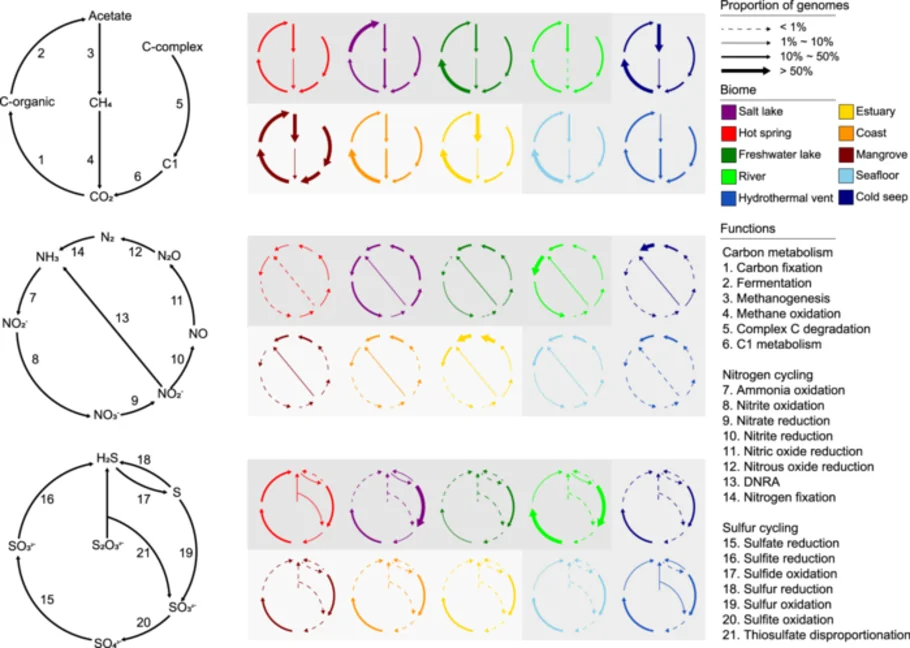
Schematic diagrams depicting the carbon, nitrogen, and sulfur cycles within archaeal communities in different sediment habitats. The color of lines stands for different biomes. The thickness of the lines indicates the proportion of genomes that are putatively involved in each metabolic process within the archaeal community. Dashed lines indicate proportions lower than 1%. DNRA, dissimilatory nitrate reduction to ammonium.

**FIGURE 4:**
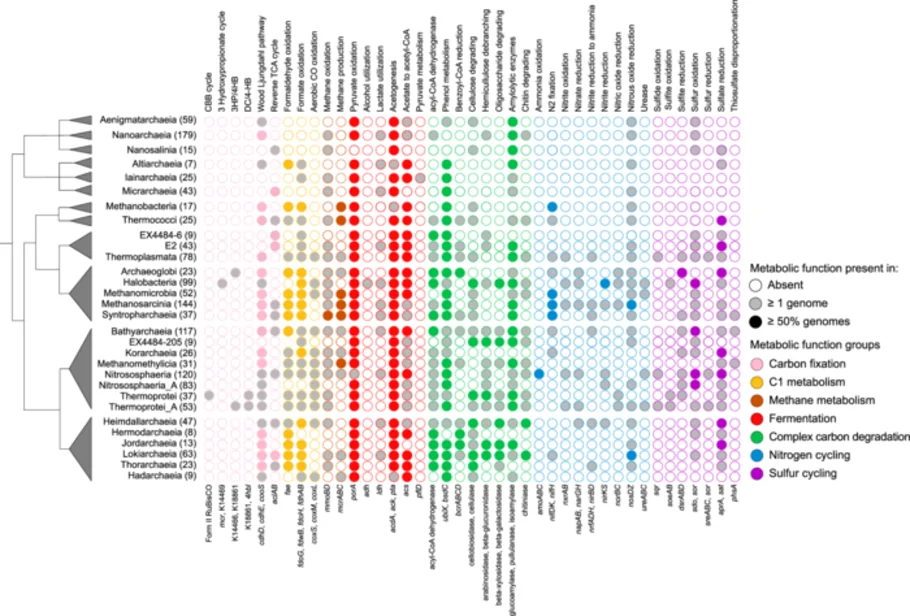
Metabolic potential of sediment archaea at the class level. The phylogenomic tree on the left was collapsed according to Figure 1D. For visualization and simplicity, genomes belonging to the same phylum were collapsed. Numbers in parentheses describe the quantity of genomes within the corresponding class. White circles indicate a lack of pathways. Gray circles represent at least one genome encoding genes within a given lineage, while colored circles signify instances where the occurrence frequency of genes in a specified lineage exceeds 50%. RuBisCO, ribulose‐1,5‐bisphosphate carboxylase/oxygenase. *mcr*, methyl‐coenzyme M reductase. K14469, propionyl‐CoA synthase. K14466, K18861, and *4hbl*, 4‐hydroxybutyrate‐‐‐CoA ligase (ADP‐forming). *cdhDE*, acetyl‐CoA decarbonylase/synthase subunit delta and gamma. *cooS*, anaerobic carbon‐monoxide dehydrogenase catalytic subunit. *aclAB*, ATP‐citrate lyase alpha‐ and beta‐subunits. *fae*, 5,6,7,8‐tetrahydromethanopterin hydro‐lyase. *fdoG*, formate dehydrogenase major subunit. *fdwB*, formate dehydrogenase beta subunit. *fdoH*, formate dehydrogenase iron‐sulfur subunit. *fdhAB*, formate dehydrogenase (coenzyme F420) subunit alpha and beta. *coxSML*, aerobic carbon‐monoxide dehydrogenase small, medium, and large subunits. *mmoBD*, methane monooxygenase regulatory protein B and component D. *mcrABC*, methyl‐coenzyme M reductase alpha, beta, and C subunits. *porA*, pyruvate ferredoxin oxidoreductase alpha subunit. *adh*, alcohol dehydrogenase. *ldh*, l‐lactate dehydrogenase. *acdA*, acetyl coenzyme A synthetase (ADP forming), alpha domain. *ack*, acetate kinase. *pta*, phosphate acetyltransferase. *acs*, acetyl‐CoA synthetase. *pflD*, formate C‐acetyltransferase. *ubiX*, flavin prenyltransferase. *bsdC*, vanillate/4‐hydroxybenzoate decarboxylase subunit C. *bcrABCD*, benzoyl‐CoA reductase subunit A, B, C, and D. *amoABC*, ammonia monooxygenase subunit A, B, and C. *nifDK*, nitrogenase molybdenum‐iron protein alpha and beta chains. *nifH*, nitrogenase iron protein. *nxrAB*, nitrate reductase/nitrite oxidoreductase subunit alpha and beta. *napA*, periplasmic nitrate reductase. *napB*, cytochrome c‐type protein. *narGH*, nitrate reductase/nitrite oxidoreductase subunit alpha and beta. *nrfADH*, cytochrome c nitrite reductase. *nirBD*, nitrite reductase (NADH) large and small subunits. *nirKS*, nitrite reductase (NO‐forming). *norBC*, nitric oxide reductase subunit B and C. *nosZ*, nitrous‐oxide reductase. *nosD*, nitrous oxidase accessory protein. *ureABC*, urease subunit gamma, beta, and alpha. *fccB*, flavocytochrome c sulfide dehydrogenase, flavin‐binding. *sqr*, sulfide:quinone oxidoreductase. *soeAB*, sulfite dehydrogenase (quinone) subunits A and B. *dsrABD*, dissimilatory sulfite reductase subunit alpha, beta, and delta. *sdo*, sulfur dioxygenase. *sor*, sulfur oxygenase/reductase. *sreA*, sulfur reductase molybdopterin subunit. *sreB*, sulfur reductase FeS subunit. *sreC*, sulfur reductase membrane anchor. *aprA*, adenylylsulfate reductase subunit A. *sat*, sulfate adenylyltransferase. *phsA*, thiosulfate reductase/polysulfide reductase chain A.

The high abundance and diversity of archaea underpin their pivotal roles in sedimentary biogeochemical cycles. In this review, we have leveraged 1543 high‐quality archaeal representative genomes to predict the metabolic potentials of sediment‐dwelling archaea (Tables S1, S2). Our metagenomic analysis has unveiled the metabolic capabilities of various archaeal lineages across diverse habitats, with a particular emphasis on carbon, nitrogen, and sulfur metabolism (Tables S3, S4). The schematic diagrams in Figure 3 depict the carbon, nitrogen, and sulfur cycles within archaeal communities in different sedimentary habitats, while Figure 4 summarizes the presence or absence of key metabolic processes across each archaeal lineage. The following sections delve into the biogeochemical cycles of sedimentary archaea, integrating genomic analyses with the latest literatures.

### Carbon metabolism

Carbon fixation processes are prevalent among sedimentary archaeal communities, especially those at land–sea interfaces (Figure 3). The distribution of archaeal carbon fixation mechanisms reflects their evolutionary status and ecological niches. As the corpus of genomic data and experimental validations grows, it becomes increasingly evident that the metabolic landscape of archaeal carbon fixation is more diverse than previously recognized. The reductive acetyl‐CoA pathway (Wood‐Ljungdahl pathway, WL) and the reductive citric acid cycle (rTCA) are predominant within archaeal communities [85], whereas other carbon fixation pathways are restricted to specific lineages (Figure 4). The 3‐hydroxypropionate/4‐hydroxybutyrate cycle (3HP/4HB) is exclusive to aerobic members of the class Archaeoglobi and Thermoprotei_A, while the 3HP and dicarboxylic acid/4‐hydroxybutyrate (DC/4HB) cycles are characteristic of thermophilic and halophilic lineages (Figure 4). Notably, AOA possesses a modified HP/HB pathway, which is considered the most energy‐efficient aerobic carbon fixation pathway [86]. Intriguingly, members of Lokiarchaeia contain all genes necessary for the DC/HB cycle, suggesting a potential for autotrophic growth [85]. Moreover, the ability to convert C1 compounds into organic products is widespread among Asgardarchaeota, Halobacteriota, and Thermoproteota (Figure 4). Further research is warranted to confirm the functional capacity of these pathways, as current insights are primarily derived from metagenomic data.

Sediments from mangroves, estuaries, and cold seeps may contain higher proportions of archaeal lineages associated with methanogenesis compared to other habitats (Figure 3). Methanogens convert substrates such as acetate, H_2_–CO_2_, and methyl compounds into methane in anaerobic sediments. Recent genomic studies have revealed the presence of the methyl‐coenzyme M reductase (*mcrA*) in various lineages, including Korarchaeia [87], Nitrososphaeria [88], Verstraetearchaeota (reclassified as the class Methanomethylicia) [89], Nezhaarchaeales [90], and Helarchaeales [40]. Mesocosm experiments have further confirmed the methanogenic capabilities of these nontraditional methanogens, including Nezhaarchaeales and Korarchaeia [91, 92], underscoring the vast diversity of methanogenic groups yet to be discovered. Archaeal methanotrophs, found in various environments (Figure 3), are key drivers of Earth's carbon flux, serving as essential biological sinks for methane. ANMEs can anaerobically convert methane to carbon dioxide, potentially mitigating the greenhouse effect in benthic sediments [93]. Additionally, the ability to utilize methyl compounds is widespread in Asgardarchaeota, including the recently proposed Sifarchaeia and Freyarchaeia, as well as the newly defined Atabeyarchaeia, suggesting their significant roles in regulating methane flux in sediments [94, 95].

Fermentation, encompassing a series of biochemical reactions such as hydrolysis, acidogenesis, acetogenesis, and methanogenesis, involves the degradation of complex carbon compounds into simpler molecules like acetate, ethanol, and CO_2_ by microbes. The capacity for fermentation and organic compound degradation is widespread among diverse archaeal lineages across various environments (Figures 3, 4). This fermentative potential is particularly pronounced among members of Asgardarchaeota, which likely utilize complex carbohydrates and detrital proteins for heterotrophic growth [69, 96]. Recent metagenomic studies have underscored the fermentative capabilities of dominant lineages such as Bathyarchaeia, Thermoprofundales, and Theionarchaea in anaerobic coastal and seafloor sediments, suggesting their role in degrading organic carbon substrates to produce acetate and alcohol [70, 97, 98]. Notably, thermophilic acetogens like EX4484‐205 [71] and Hydrothermarchaeia [76] may play essential roles in transforming various carbon substrates in geothermal habitats, providing insights into the early evolution of life.

### Nitrogen metabolism

Nitrogen fixation, a pivotal process in the nitrogen cycle, is commonly observed in various sediment habitats (Figure 3) and can convert N_2_ into bioavailable nitrogen compounds. Nitrogen gas in sediments represents the end product of denitrification. In contrast to bacteria, the distribution of archaeal diazotrophs is relatively limited, being predominantly confined to methanogenic and sulfate‐reducing lineages (Figure 4). Recent genomic studies have identified the presence of *nifH* (nitrogenase iron protein) in Bathyarchaeia, Archaeoglobi, Methanomethylicia, Theionarchaea, and certain Asgardarchaeota in benthic sediments [61, 99], suggesting that the potential for nitrogen fixation may extend across broader archaeal lineages, providing a crucial mechanism for alleviating nitrogen limitation in diverse ecosystems.

Nitrification significantly impacts the availability of inorganic nitrogen in ecosystems. Microbial ammonia oxidation, the first and rate‐limiting step of nitrification, is primarily mediated by AOA, ammonia‐oxidizing bacteria (AOB), and complete ammonia oxidizers (comammox bacteria). To date, all identified AOA belong to the class Nitrososphaeria, which are considered major contributors to ammonia oxidation across various environments, likely due to their low oxygen requirements, broad salinity tolerance, and high cellular affinity for ammonia [100, 101, 102]. Key genes encoding nitrite oxidoreductase (*nxrAB*) have been detected in members of Halobacteria isolated from salt lake sediments (Figures 3, 4) [103]. Notably, nitrite oxidoreductase homologues have been identified in members of Nitrososphaerales residing in terrestrial subsurface aquifers, potentially acquired through horizontal gene transfer from co‐occurring bacteria [104].

Conversely, denitrification, which converts oxidized inorganic nitrogen compounds (NO_2_^−^ and NO_3_^−^) back to atmospheric N₂ gas, is crucial for nitrogen removal in sediments. Denitrifying archaea use nitrate or nitrite as an electron acceptor for anaerobic respiration, resulting in the generation of gaseous NO, N_2_O, and N_2_. Archaeal denitrification is commonly identified in thermophilic and halophilic lineages (Figure 4) [105]. Members of AOA have been shown to encode nitrite reductases (*nirK*), indicating their potential to produce N_2_O through the proposed nitrifier‐denitrification pathway [106]. Recent investigations suggest that Thorarchaeia, Heimdallarchaeia, and Thermoprofundales may also participate in nitrite or nitrate reduction in benthic sediments [40, 70, 107]. However, further evidence for gene expression is essential to validate their significance in nitrogen turnover within sediments.

### Sulfur metabolism

The oxidation of sulfur substrates represents a major energy acquisition strategy prevalent in extreme environments (Figure 3). Key genes encoding sulfur dioxygenase (*sdo*) and sulfur oxygenase/reductase (*sor*) involved in sulfur oxidation processes are widely distributed among Halobacteriota and Thermoplasmatota from salt lake sediments (Figure 4). Additionally, representatives of Heimdallarchaeia and Bathyarchaeia may also participate in sulfur oxidation in seafloor and coastal sediments (Figure 3). Recent studies have demonstrated that Caldarchaeales (formerly classified as Aigarchaeota) and Nitrososphaerales can perform sulfur oxidation in hot spring sediments [61, 108, 109]. In hydrothermal vents, Hydrothermarchaeia are considered important mediators of sulfide and thiosulfate oxidation processes [76].

Conversely, the reduction of sulfur compounds is more commonly observed across various archaeal lineages. Most thermophilic Thermoprotei and Archaeoglobi harbor genes associated with adenylylsulfate reductase (*aprA*), sulfate adenylyltransferase (*sat*), and dissimilatory sulfide reductases (*dsrABD*) (Figure 4). In geothermal environments, Caldarchaeales, Pacearchaeales, and Woesearchaeales may perform dissimilatory sulfite reduction [108, 110], while EX4484‐205 may reduce sulfur using H_2_ or organic substrates to produce H_2_S [71]. Notably, certain Korarchaeia members contain methyl‐coenzyme M reductase and dissimilatory sulfide reductase‐related genes, indicating their potential to couple sulfur reduction with anaerobic methane oxidation in hot springs [87]. Coincidentally, Hydrothermarchaeia may couple nitrate reduction with the oxidation of reduced sulfur compounds as an energy‐generating process in hydrothermal vents [111]. Halophilic Halobacteria have been shown to produce dimethylsulfide through the dissimilatory reduction of dimethylsulfoxide [112], playing vital roles in transforming intermediate sulfur compounds in salt lakes. The sulfate reduction process may also be carried by widespread Bathyarchaeia and Nitrososphaeria in river, coastal, and seafloor sediments (Figures 2, 4). Particularly in coastal regions, Theionarchaea are likely to contribute significantly to the reduction of benthic polysulfide and thiosulfate [98]. However, it is necessary to verify the specific contribution of sediment archaea in situ, as most lineages encode genes associated with both reductase and oxidase of sulfur substrates (Figure 4), to obtain a more comprehensive understanding of the sediment sulfur cycle contributed by archaea.

## 4 OUTLOOKS

In the genomic era, our understanding of archaeal biology has advanced significantly due to the development of culture‐independent technologies, highlighting their diversity, distribution, metabolic potentials, and ecological roles. However, while some novel groups are only identified through rRNA surveys, others remain elusive due to primer biases, suggesting that analyses reliant on limited 16S rRNA and functional genes may overlook numerous archaeal taxa. Most critically, the mere presence of these genes does not invariably indicate their activity or ecological functions, complicating the assessment of their roles in local environments. It is imperative to correlate in situ physicochemical parameters with genomic data. Techniques such as DNA stable isotope probing, metatranscriptomics, metabolomics, and metaproteomics aid in functional predictions, while methods like rRNA‐targeted fluorescent in situ hybridization and bioorthogonal noncanonical amino acid tagging fluorescence‐activated cell sorting elucidate cell activity. The integration of community‐level genomic reconstruction with activity assays can clarify microbial diversity and metabolic mechanisms. Conversely, many hypotheses regarding archaeal physiology and evolution necessitate validation through culture‐based experiments, which have historically proven challenging. Recent innovations, including coculture, direct interspecies electron transfer, single‐cell isolation, and high‐throughput culturing, have improved archaeal cultivation, potentially enabled the isolation of previously uncultured archaea and facilitated crucial physiological research.

Recent research hotspots on archaea have broadened significantly, focusing not only on their diversity and metabolic functions but also on the role of archaeal viruses and the origins of eukaryotes. Archaeal viruses represent some of the most intriguing entities within the virosphere, characterized by their distinct morphotypes and genome compositions. The remarkable diversity of archaeal virions, featuring unique morphotypes not observed in bacteriophages or eukaryotic viruses, has been highlighted by metagenomics, single‐cell genomics, and genome mining. These studies have significantly advanced our understanding of the archaeal virosphere, emphasizing its crucial ecological roles in microbial community dynamics and global nutrient cycling. For instance, virus‐mediated lysis of archaea, particularly in deep‐ocean environments, plays a pivotal role in carbon turnover, contributing substantially to the annual carbon release from deep‐sea sediments at the gigaton scale. Furthermore, the origin of the eukaryotic cell remains a significant debate in modern biology. The recognition of the Archaea domain has significantly enriched our understanding of life's vast diversity, with recent discoveries of new Asgard lineages revealing various ESPs involved in cytoskeleton formation, transport, translation, transcription, and degradation pathways. These findings support symbiogenic models that propose eukaryotes originated from an archaeal host.

## 5 CONCLUSION

In summary, the discovery of archaea has catalyzed evolutionary revolutions pertinent to cellular life. Subsequent explorations of archaeal diversity and metabolic potentials have underscored their pivotal roles in biogeochemical cycles. While culture‐independent technologies have advanced our research on most archaeal groups, cultivation and long‐term enrichment experiments are essential for verifying inferred metabolic capacities and functions. Moreover, modeling and long‐term monitoring analyses are necessary to elucidate the intricate relationships and interactions between archaea, other microbes, viruses, and humans. Achieving a comprehensive understanding of archaeal biology necessitates a multidisciplinary approach and the integration of diverse methodologies.

## 6 METHODS

### Genomic data collection

To illustrate and compare the archaeal diversity across different sediment habitats, we amassed a collection of representative archaeal genomes from the publicly available data within the GTDB database. The metadata, encompassing details of the isolation source, sample location, and fundamental genome characteristics for each archaeal genome, was extracted from the GTDB online repository (https://data.gtdb.ecogenomic.org/releases/release220/). Briefly, within the 12,447 representative archaeal genomes (GTDB Release 220), a subset of 2063 species‐level genomes was selected for subsequent analysis based on the following criteria: (i) the isolation sources were identified as one of the natural sediment types previously mentioned; (ii) the genomes were accompanied by spatial location data, including latitude and longitude; (iii) the genomes were of medium to high quality, defined as having a completeness of ≥50% and contamination ≤10% (Table S1). These 2,063 archaeal representative genomes, which are globally distributed across diverse sedimentary environments including inland hot springs, salt lakes, freshwater lakes, rivers, mangroves, estuaries, coastal regions, seafloor, cold seeps, and hydrothermal vents (Table S1 and Figure 1A), represent 18 phyla, 52 classes, 120 orders, 331 families, and 769 genera. Furthermore, to more precisely delineate the phylogeny and metabolic functions of these genomes, a subset of 1543 high‐quality genomes (completeness ≥ 80% and contamination ≤ 10%), affiliated with 16 phyla and 48 classes, was utilized for phylogenetic analysis and functional annotation (Figure 1B).

### Functional annotations

The multiple sequence alignments of 53 concatenated conserved archaeal marker genes from the 1543 high‐quality genomes were retrieved using GTDB‐Tk (v.2.4.0) [113]. The poorly aligned regions were removed using trimAl (v.1.4. rev22) with the parameter: ‐automated1 [114]. Phylogeny was inferred using IQ‐TREE (v.2.3.6) with 1000 ultrafast bootstrapping iterations [115]. The best model of LG + F + G4 was determined using ModelFinder, which is well supported by Bayesian information criterion. The phylogenetic tree was visualized using iTOL (v.6) [116].

Functional annotation of these genomes was conducted using METABOLIC‐G (v.4.0) under default settings [117]. Briefly, gene calling was performed for each genome using Prodigal with “‐p single” option to identify open reading frames (ORFs) [118]. The ORFs were subsequently searched against the KEGG, TIGRfam, Pfam and collected HMM profiles associated with key biogeochemical cycling using HMMER (v.3.3.2) [119]. The presence or absence of major metabolic pathways involved in carbon, nitrogen, and sulfur cycles across the 1543 genomes was summarized (Table S2). The prevalence of carbon, nitrogen, and sulfur‐related metabolic pathways within the archaeal communities inhabiting each biome was illustrated and compared (Table S3). Additionally, the number of genomes involved in these cycles was tallied for the top 30 archaeal classes with the highest number of representative genomes (Table S4).

## AUTHOR CONTRIBUTIONS

**Dayu Zou**: Conceptualization; methodology; writing—original draft; writing—review and editing. **Yanling Qi**, **Jinjie Zhou**, and **Yang Liu**: Writing—review and editing. **Meng Li**: Writing—review and editing; Conceptualization; funding acquisition; writing—original draft.

## CONFLICT OF INTEREST STATEMENT

The authors declare no conflicts of interest.

## ETHICS STATEMENT

No animals or humans were involved in this study.

## Supporting information


**Table S1:** The basic information of sediment archaeal representative genomes used in this review, including the accession numbers, taxonomy, biome, and genome quality.
**Table S2:** The presence and absence of major genes and pathways associated with carbon, nitrogen, and sulfur metabolisms of the 1543 high‐quality archaeal representative genomes.
**Table S3:** The number genomes encoded each carbon, nitrogen, and sulfur related metabolic pathway of the archaea communities from different sediment habitats.
**Table S4:** The number of representative genomes involved in each of the carbon, nitrogen, and sulfur metabolic pathway for the top 30 archaeal classes.

## Data Availability

Data sharing is not applicable to this article as no datasets were generated or analyzed during the current study. No new data and scripts were generated in this review. The sediment archaeal representative genomes used in this review are publicly available in the GTDB repository, which are included in the latest release version 220 (https://data.gtdb.ecogenomic.org/releases/release220/). Supplementary materials (tables, graphical abstract, slides, videos, Chinese translated version, and update materials) may be found in the online DOI or iMeta Science http://www.imeta.science/imetaomics/.
